# Lardner’s Natural Philosophy

**Published:** 1853-05

**Authors:** 


*Lardner's Natural Philosophy*, Second course, embracing Heat, Magnetism, Common Electricity, Voltaiic Electricity. Philadelphia, Blanchard & Lea, 1853.

The popular works of Dr. Lardner, are too well known to need recommendation. Astronomy and meteorology will form the third and concluding course. The whole will constitute a compendious encyclopedia of physical and astronomical science. For sale by our booksellers generally. J.

